# Supplementary material for: OpenMM 7: Rapid development of high performance algorithms for molecular dynamics
Source: PLoS Comput Biol. 2017 Jul 26;13(7):e1005659. doi: 10.1371/journal.pcbi.1005659 (PMC5549999; doi:10.1371/journal.pcbi.1005659)
Supplement: S1 Examples — (DOCX) [file pcbi.1005659.s003.docx]

Using CHARMM Force Fields and CHARM-GUI With OpenMM

OpenMM scripts can directly read CHARMM input files. This enables the use of all the powerful setup tools in the CHARMM ecosystem that a user might be familiar with such as [CHARMM-GUI](http://onlinelibrary.wiley.com/doi/10.1002/jcc.20945/abstract), [VMD](http://www.sciencedirect.com/science/article/pii/0263785596000185?via%3Dihub), [CGenFF](https://cgenff.paramchem.org/), etc. This allows users who are already working in the CHARMM environment to harness the GPU speeds that OpenMM provides without having to modify their simulation system description files. It also allows them to use CHARMM force fields that are not already included in OpenMM such as the general CHARMM force field (CGenFF).

This example demonstrates how to use CHARMMM files that were generated with CHARMM-GUI in an OpenMM script. The OpenMM Python layer includes several classes to load CHARMM files. The CharmmPsfFile class reads the PSF file and instantiates a chemical structure. One can then call the createSystem() method on it to create an OpenMM system object. For the atomic coordinates, a regular PDB file can be used, or the CharmmCrdFile or CharmmRstFile classes can be used to read CHARMM coordinate files. Files containing force field definitions come in a variety of formats such as prm, par, top, rtf, inp and str. These files are loaded into a CharmmParameterSet object, which is then included as the first parameter when createSystem() is called on the chemical structure. For this example, the [membrane builder](http://dx.doi.org/10.1371/journal.pone.0000880) in CHARMM-GUI was used to generate the input files for the B2AR in a POPC lipid membrane. The membrane builder provides a straightforward way to go from the RCSB X-ray structure to the protein embedded in a membrane with all the relevant CHARMM input files.

from simtk.openmm.app import *

from simtk.openmm import *

from simtk.unit import *

from sys import stdout, exit, stderr

# Load CHARMM files

psf = CharmmPsfFile('step5_charmm2omm.psf')

pdb = PDBFile('step5_charmm2omm.pdb')

params = CharmmPsfFile('par_all36_prot.rtf', 'top_all36_prot.prm',

'par_all36_lipid.rtf', 'top_all36_lipid.prm',

'toppar_water_ion.str')

# Create an OpenMM system by calling createSystem on psf

system = psf.createSystem(params, nonbondedMethod=NoCutoff,

nonbondedCutoff=1*nanometer, constraints=HBonds)

integrator = LangevinIntegrator(300*kelvin, # Temperature of head bath

1/picosecond, # Friction coefficient

0.002*picoseconds) # Time step

simulation = Simulation(psf.topology, system, integrator)

simulation.context.setPositions(pdb.positions)

simulation.minimizeEnergy()

# Set up the reporters to report energies every 1000 steps.

simulaiton.reporters.append(PDBReporter('output.pdb', 1000))

simulation.reporters.append(StateDataReporter(stdout, 1000, step=True,

potentialEnergy=True, temperature=True))

# run simulation

simulation.step(10000)

CHARMM-GUI also generates a more elaborate set of OpenMM scripts to run equilibration and the production simulation that are very straightforward to use. A tutorial that walks through this process is provided [here](https://github.com/ChayaSt/openmm7tutorials/tree/master/b2ar_membrane). When OpenMM is selected in the last step, CHARMM-GUI provides all the relevant OpenMM scripts in the downloaded file. The parameters and arguments for OpenMM objects and functions are provided in the inp files for all equilibration steps and production. This makes it simple to change parameters such as the time step or electrostatic cutoff method.

Using Amber Force Fields With OpenMM

OpenMM allows users to model their systems using Amber and provide prmtop and inpcrd files as input. This allows users more familiar with the Amber modeling environment to continue using their setup tools, while harnessing the speed and versatility of OpenMM. This also allows the use of non-standard force fields that have been published for use with Amber, such as metal ion models where dummy atoms are applied to mimic particular coordination geometries. Furthermore, this is facilitated by OpenMM's support for extra particles—particles that are not ordinary atoms, such as these metal dummy atoms, virtual sites in many water models, etc.

This example illustrates the use of Amber's tleap to set up a simulation of the histone methyltransferase SETD2 (Uniprot: [Q9BYW2](http://www.uniprot.org/uniprot/Q9BYW2)), including three structural Zn2+ cations described with the tetrahedral Cationic Dummy Atom Approach (CADA: [DOI:10.1007/s008940050119](https://dx.doi.org/10.1007/s008940050119), [Pang lab](http://www.mayo.edu/research/labs/computer-aided-molecular-design/projects/zinc-protein-simulations-using-cationic-dummy-atom-cada-approach)).

In addition to tleap we use two other programs, [PDBFixer](https://github.com/pandegroup/pdbfixer) and [MDTraj](http://mdtraj.org/), to prepare the files for simulating. Rather than running each program separately, we use a Python script to automate the whole process. This helps reproducibility.

We begin from the 4H12 PDB file, and use PDBFixer to add missing residues (only those in the middle of the chain) and missing heavy atoms. The CADA model requires particular naming of residues in the PDB file (e.g. renaming the ligating cysteines from CYS to CYM), so we use MDTraj to make these changes and to remove unwanted water and ligand residues. Finally, tleap is run to add hydrogens and the Zn2+ dummy atoms, and to parametrize the system. We use the ff99SBildn force field and znb.lib, and frcmod.zinc files from the CADA model (downloaded from [Pang lab](http://www.mayo.edu/research/labs/computer-aided-molecular-design/projects/zinc-protein-simulations-using-cationic-dummy-atom-cada-approach)). The prmtop and inpcrd files are saved for simulation in OpenMM.

from pdbfixer import PDBFixer

from simtk.openmm.app import PDBFile

import mdtraj as md

# clean up the original PDB file and add missing residues and heavy atoms

fixer = PDBFixer('pdb4h12.ent')

fixer.findMissingResidues()

# only add missing residues in the middle of the chain, do not add terminal ones

chains = list(fixer.topology.chains())

keys = fixer.missingResidues.keys()

missingResidues = dict()

for key in keys:

chain = chains[key[0]]

if not (key[1] == 0 or key[1] == len(list(chain.residues()))):

missingResidues[key] = fixer.missingResidues[key]

fixer.missingResidues = missingResidues

fixer.findMissingAtoms()

fixer.addMissingAtoms()

PDBFile.writeFile(fixer.topology, fixer.positions, open('4h12_fixed.pdb', 'w'))

# keep only protein and zinc ions

traj = md.load('4h12_fixed.pdb')

traj = traj.atom_slice(traj.top.select('(protein and not resname SAH) or resname ZN'))

# implement changes necessary for the use of the dummy atom Zn2+ model

# change residue name of the zincs from ZN to ZNB, and atom names from ZN to Zn

for residue in traj.top.chain(1).residues:

residue.name = 'ZNB'

for atom in traj.top.chain(1).atoms:

atom.name = 'Zn'

# change name of cysteines coordinating zincs to CYM (deprotonated cysteine)

for residue in traj.top.chain(0).residues:

if residue.index in [86, 92, 82, 69, 54, 52, 73, 184, 233, 238, 231]:

residue.name = 'CYM'

traj.save('4h12_fixed_protein_zn_only.pdb')

# save the tleap script to file

with open('leaprc.setd2', 'w') as f:

f.write('''

source oldff/leaprc.ff99SBildn

addAtomTypes { { "DZ" "Zn" "sp3" } { "Zn" "Zn" "sp3" } }

loadOff znb.lib

loadamberparams frcmod.zinc

x = loadPdb 4h12_fixed_protein_zn_only.pdb

addIons x Cl- 0

solvateBox x TIP3PBOX 10.0

savePdb x topology.pdb

saveAmberParm x input.prmtop input.inpcrd

quit

''')

# run tleap

os.system('tleap -f leaprc.setd2')

To run the simulation, we load the prmtop and inpcrd files by creating AmberPrmtopFile and AmberInpcrdFile objects. Next, the System is created by calling the createSystem() method on the AmberPrmtopFile object. Next, the LangevinIntegrator and the Simulation are set up, using the topology from the AmberPrmtopFile and positions from the AmberInpcrdFile. In this example, we use the CUDA platform with mixed precision. The simulation is energy minimized and equilibrated for 100 steps. Reporters are attached and the production simulation propagated for 50 ns.

from __future__ import print_function

from simtk.openmm import app

import simtk.openmm as mm

from simtk import unit

from sys import stdout

# load in Amber input files

prmtop = app.AmberPrmtopFile('input.prmtop')

inpcrd = app.AmberInpcrdFile('input.inpcrd')

# prepare system and integrator

system = prmtop.createSystem(nonbondedMethod=app.PME,

nonbondedCutoff=1.0*unit.nanometers, constraints=app.HBonds, rigidWater=True,

ewaldErrorTolerance=0.0005)

integrator = mm.LangevinIntegrator(300*unit.kelvin, 1.0/unit.picoseconds,

2.0*unit.femtoseconds)

integrator.setConstraintTolerance(0.00001)

# prepare simulation

platform = mm.Platform.getPlatformByName('CUDA')

properties = {'CudaPrecision': 'mixed'}

simulation = app.Simulation(prmtop.topology, system, integrator, platform,

properties)

simulation.context.setPositions(inpcrd.positions)

# minimize

print('Minimizing...')

simulation.minimizeEnergy()

# equilibrate for 100 steps

simulation.context.setVelocitiesToTemperature(300*unit.kelvin)

print('Equilibrating...')

simulation.step(100)

# append reporters

simulation.reporters.append(app.DCDReporter('trajectory.dcd', 1000))

simulation.reporters.append(app.StateDataReporter(stdout, 1000, step=True,

potentialEnergy=True, temperature=True, progress=True, remainingTime=True,

speed=True, totalSteps=25000000, separator='\t'))

# run 50 ns of production simulation

print('Running Production...')

simulation.step(25000000)

print('Done!')

Simulating Drude Particles

This example illustrates the use of the CHARMM polarizable force field. This force field introduces some complications beyond the scripts shown in the main text. First, it uses various kinds of "extra particles" to represent special interaction sites: Drude particles to allow polarization and virtual sites to represent lone pairs. These extra particles must be added to the molecular topology before it can be simulated.

This is done using the Modeller class. To use it, one provides an initial topology and atomic positions, then calls methods on it to modify them in a variety of ways. This example uses the addExtraParticles() method, which adds whatever particles are needed for a given force field. The same method can be used for other force fields that require extra particles, such as the TIP4P or TIP5P water models.

Second, special integration methods are needed to simulate systems with Drude particles. For this example, we use a DrudeSCFIntegrator. This is an integrator that first computes the atom positions based on the equations of motion, then performs an energy minimization to select positions for the Drude particles.

from simtk.openmm.app import *

from simtk.openmm import *

from simtk.unit import *

pdb = PDBFile('input.pdb')

forcefield = ForceField('charmm_polar_2013.xml')

modeller = Modeller(pdb.topology, pdb.positions)

modeller.addExtraParticles(forcefield)

system = forcefield.createSystem(modeller.topology, nonbondedMethod=PME,

nonbondedCutoff=1*nanometer, constraints=HBonds)

integrator = DrudeLangevinIntegrator(300*kelvin, 1/picosecond, 1*kelvin,

1/picosecond, 0.002*picoseconds)

simulation = Simulation(modeller.topology, system, integrator)

simulation.context.setPositions(modeller.positions)

simulation.context.setVelocitiesToTemperature(300*kelvin)

simulation.reporters.append(DCDReporter('output.pdb', 1000))

simulation.reporters.append(StateDataReporter('output.log', 1000, time=True, potentialEnergy=True, temperature=True))

simulation.step(1000000)

Alchemical Free Energy

OpenMM's custom forces—which allow the programmer to express a potential algebraically, potentially with multiple parameters that can be adjusted on the fly—allow a great deal of flexibility and simplicity in encoding potentials while still achieving high performance on GPUs. One common use of this facility is to convert standard interactions (such as Lennard-Jones potentials) into alchemically-modified potentials for the purposes of computing free energy differences. The alchemical free energy code [YANK](http://github.com/choderalab/yank), for example, uses a variety of custom forces to represent alchemically-modified potentials for [protein-ligand alchemical binding free energy calculations](http://dx.doi.org/10.1007/s10822-013-9689-8).

As a simple example of how this is facilitated by custom forces, consider computing the chemical potential of liquid argon by estimating the free energy of alchemically annihilating a Lennard-Jones particle. First, we create a simple Lennard-Jones fluid to represent liquid argon at 120 K and 80 atm, which can be conveniently done using the testsystems module of the conda-installable [openmmtools](http://github.com/choderalab/openmmtools) package:

from simtk import openmm, unit

# Create a Lennard-Jones fluid

pressure = 80*unit.atmospheres

temperature = 120*unit.kelvin

collision_rate = 5/unit.picoseconds

timestep = 2.5*unit.femtoseconds

from openmmtools.testsystems import LennardJonesFluid

sigma = 3.4*unit.angstrom; epsilon = 0.238 * unit.kilocalories_per_mole

fluid = LennardJonesFluid(sigma=sigma, epsilon=epsilon)

[system, positions] = [fluid.system, fluid.positions]

# Add a barostat

barostat = openmm.MonteCarloBarostat(pressure, temperature)

system.addForce(barostat)

To allow one of the Lennard-Jones particles to be alchemically eliminated, we create a [CustomNonbondedForce](http://docs.openmm.org/7.1.0/api-python/generated/simtk.openmm.openmm.CustomNonbondedForce.html#simtk.openmm.openmm.CustomNonbondedForce) that will compute the interactions between the alchemical particle and the remaining chemical particles using a [softcore potential](http://dx.doi.org/10.1016/0009-2614%2894%2900397-1). The alchemically-modified particle has its Lennard-Jones well depth (epsilon parameter) set to zero in the original NonbondedForce, while the CustomNonbondedForce is set to evaluate only the interactions between the alchemically-modified particle and the remaining particles using [addInteractionGroup()](http://docs.openmm.org/7.1.0/api-python/generated/simtk.openmm.openmm.CustomNonbondedForce.html#simtk.openmm.openmm.CustomNonbondedForce.addInteractionGroup) to specify only interactions between these groups are to be computed. A global context parameter lambda is created to control the coupling of the alchemically-modified particle with the rest of the system during the simulation. The Lennard-Jones parameters sigma and epsilon are implemented as per-particle parameters, though this is not strictly necessary in this case since all particles are equivalent.

# Retrieve the NonbondedForce

forces = { force.__class__.__name__ : force for force in system.getForces() }

nbforce = forces['NonbondedForce']

# Add a CustomNonbondedForce to handle only alchemically-modified interactions

alchemical_particles = set([0])

chemical_particles = set(range(system.getNumParticles())) - alchemical_particles

energy_function = 'lambda*4*epsilon*x*(x-1.0); x = (sigma/reff_sterics)^6;'

energy_function += 'reff_sterics = sigma*(0.5*(1.0-lambda) + (r/sigma)^6)^(1/6);'

energy_function += 'sigma = 0.5*(sigma1+sigma2); epsilon = sqrt(epsilon1*epsilon2);'

custom_force = openmm.CustomNonbondedForce(energy_function)

custom_force.addGlobalParameter('lambda', 1.0)

custom_force.addPerParticleParameter('sigma')

custom_force.addPerParticleParameter('epsilon')

for index in range(system.getNumParticles()):

[charge, sigma, epsilon] = nbforce.getParticleParameters(index)

custom_force.addParticle([sigma, epsilon])

if index in alchemical_particles:

nbforce.setParticleParameters(index, charge*0, sigma, epsilon*0)

custom_force.addInteractionGroup(alchemical_particles, chemical_particles)

system.addForce(custom_force)

We then create a [LangevinIntegrator](http://docs.openmm.org/7.1.0/api-python/generated/simtk.openmm.openmm.LangevinIntegrator.html#simtk.openmm.openmm.LangevinIntegrator) and [Context](http://docs.openmm.org/7.1.0/api-python/generated/simtk.openmm.openmm.Context.html#simtk.openmm.openmm.Context) to run the simulation, and run a series of simulations at different values of lambda by using [context.setParameter()](http://docs.openmm.org/7.1.0/api-python/generated/simtk.openmm.openmm.Context.html#simtk.openmm.openmm.Context.setParameter) to update the alchemical parameter on the fly. For each configuration sample that is collected, we can easily scan through the energy at different lambda values by simply alternating between context.setParameter() to update lambda and context.getState() to retrieve potential energies at the new alchemical state.

# Create a context

integrator = openmm.LangevinIntegrator(temperature, collision_rate, timestep)

context = openmm.Context(system, integrator)

context.setPositions(positions)

# Minimize energy

print('Minimizing energy...')

openmm.LocalEnergyMinimizer.minimize(context)

# Collect data

nsteps = 2500 # number of steps per sample

niterations = 50 # number of samples to collect per alchemical state

import numpy as np

lambdas = np.linspace(1.0, 0.0, 10) # alchemical lambda schedule

nstates = len(lambdas)

u_kln = np.zeros([nstates,nstates,niterations], np.float64)

kT = unit.AVOGADRO_CONSTANT_NA * unit.BOLTZMANN_CONSTANT_kB * integrator.getTemperature()

for k in range(nstates):

for iteration in range(niterations):

print('state %5d iteration %5d / %5d' % (k, iteration, niterations))

# Set alchemical state

context.setParameter('lambda', lambdas[k])

# Run some dynamics

integrator.step(nsteps)

# Compute energies at all alchemical states

for l in range(nstates):

context.setParameter('lambda', lambdas[l])

u_kln[k,l,iteration] = context.getState(getEnergy=True).getPotentialEnergy() / kT

Finally, the [multistate Bennett acceptance ratio (MBAR)](https://dx.doi.org/10.1063%2F1.2978177) is used to estimate the free energy of annihilating the particle using the conda-installable [pymbar](http://pymbar.org/) package. In order to estimate how much data must be discarded to equilibration, we use a scheme for [automated equilibration detection](http://dx.doi.org/10.1021/acs.jctc.5b00784) and subsequent extraction of decorrelated samples found in the [pymbar.timeseries](http://github.com/choderalab/pymbar) module.

# Estimate free energy of Lennard-Jones particle insertion

from pymbar import MBAR, timeseries

# Subsample data to extract uncorrelated equilibrium timeseries

N_k = np.zeros([nstates], np.int32) # number of uncorrelated samples

for k in range(nstates):

[nequil, g, Neff_max] = timeseries.detectEquilibration(u_kln[k,k,:])

indices = timeseries.subsampleCorrelatedData(u_kln[k,k,:], g=g)

N_k[k] = len(indices)

u_kln[k,:,0:N_k[k]] = u_kln[k,:,indices].T

# Compute free energy differences and statistical uncertainties

mbar = MBAR(u_kln, N_k)

[DeltaF_ij, dDeltaF_ij, Theta_ij] = mbar.getFreeEnergyDifferences()

A downloadable version of this example is available on [github](https://github.com/choderalab/openmm7tutorials/blob/master/alchemical-free-energy). A more fully featured toolkit for re-encoding standard OpenMM forces as alchemically-modified custom forces is available via the conda-installable [alchemy](https://github.com/choderalab/alchemy) package.
